# Taxis assays measure directional movement of mosquitoes to olfactory cues

**DOI:** 10.1186/1756-3305-6-131

**Published:** 2013-05-03

**Authors:** Lena M Lorenz, Aidan Keane, Jason D Moore, Cristina J Munk, Laura Seeholzer, Antony Mseka, Emmanuel Simfukwe, Joseph Ligamba, Elizabeth L Turner, Lubandwa R Biswaro, Fredros O Okumu, Gerry F Killeen, Wolfgang R Mukabana, Sarah J Moore

**Affiliations:** 1 Disease Control Department, London School of Hygiene & Tropical Medicine, Keppel Street, London, WC1E 7HT, UK; 2 Department of Life Sciences, Imperial College London, Silwood Park Campus, Ascot, SL5 7PY, UK; 3 Biomedical and Environmental Thematic Groups, Ifakara Health Institute, PO Box 53, Ifakara, Tanzania; 4 Department of Biostatistics and Bioinformatics and Duke Global Health Institute, Duke University, Durham, NC, USA; 5 Liverpool School of Tropical Medicine, Pembroke Place, Liverpool, L3 5QA, UK; 6 School of Biological Sciences, University of Nairobi, Chiromo Road, Nairobi, Kenya

**Keywords:** Olfaction, Taxis box, Field assay, Odour, Directional movement, Disease control, Arthropods, *Anopheles*, Malaria

## Abstract

**Background:**

Malaria control methods targeting indoor-biting mosquitoes have limited impact on vectors that feed and rest outdoors. Exploiting mosquito olfactory behaviour to reduce blood-feeding outdoors might be a sustainable approach to complement existing control strategies. Methodologies that can objectively quantify responses to odour under realistic field conditions and allow high-throughput screening of many compounds are required for development of effective odour-based control strategies.

**Methods:**

The olfactory responses of laboratory-reared *Anopheles gambiae* in a semi-field tunnel and *A. arabiensis* females in an outdoor field setting to three stimuli, namely whole human odour, a synthetic blend of carboxylic acids plus carbon dioxide and CO_2_ alone at four distances up to 100 metres were measured in two experiments using three-chambered taxis boxes that allow mosquito responses to natural or experimentally-introduced odour cues to be quantified.

**Results:**

Taxis box assays could detect both activation of flight and directional mosquito movement. Significantly more (6-18%) *A. arabiensis* mosquitoes were attracted to natural human odour in the field up to 30 metres compared to controls, and blended synthetic human odours attracted 20% more *A. gambiae* in the semi-field tunnel up to 70 metres. Whereas CO_2_ elicited no response in *A. arabiensis* in the open field, it was attractive to *A. gambiae* up to 50 metres (65% attraction compared to 36% in controls).

**Conclusions:**

We have developed a simple reproducible system to allow for the comparison of compounds that are active over medium- to long-ranges in semi-field or full-field environments. Knowing the natural range of attraction of anopheline mosquitoes to potential blood sources has substantial implications for the design of malaria control strategies, and adds to the understanding of olfactory behaviour in mosquitoes. This experimental strategy could also be extended from malaria vectors to other motile arthropods of medical, veterinary and agricultural significance.

## Background

Flying and biting arthropods are economically and epidemiologically important as agricultural pests and medical and veterinary vectors of disease, with over 3.2 billion people threatened by malaria and more than 5 billion people at risk from arboviruses (e.g. dengue fever) [1]. Most of these insects, such as mosquitoes, tsetse flies and moths, rely on olfactory cues for many essential life processes: attractive odours are used to find mates, food and breeding sites, while repellent odours include plant defensive toxins and predatory signals that deter oviposition [2-4]. An insect’s responsiveness to host-emitted odours will affect its reproductive success and life-history, which in turn will determine its evolutionary fitness. A vector’s feeding success is also a strong determinant of the epidemiology and transmission of infectious diseases [5]. For example, anopheline mosquito species in sub-Saharan Africa such as *Anopheles gambiae* sensu stricto (s.s.) are some of the most efficient vectors of human malaria, precisely because their odorant receptors are narrowly tuned to compounds of human sweat [6]. Selective loss of sensitivity to such cues would have large fitness costs for the insects; thus resistance is unlikely to develop when utilizing odours as lures or repellents, which could therefore become an evolutionarily sustainable control method to complement existing strategies [7].

Behavioural responses of insects to olfactory cues for mass trapping have been exploited by entomologists for more than 200 years to manage and even eradicate a number of pest species [8-10]. More recently, mass trapping using odour-baited traps (OBTs) and lure-and-kill strategies have been suggested to control malaria vectors [11-14]. Many studies have examined short-range odour detection of anopheline mosquitoes mostly under laboratory conditions. Studies include identification of olfactory receptor neurons [6,15,16], and *in vivo* measurements of behavioural responses of insects to single odours or odour blends with electrophysiology [17], dual-choice olfactometers [18,19] and wind-tunnel experiments over distances of less than 3 metres [20,21]. Semi-field systems have been used to examine short- to medium-range taxis in large netting chambers exposed to ambient temperature and wind conditions [22]. In the field, long-range responses to odours have been tested using a series of traps or electrocuting nets that surround an attractive odour source or are themselves emitting odours [23-25]. While each of the described methodologies provides useful information about one or more aspects of odour-based taxis, there is a need for a methodology that can objectively quantify medium- to long-range responses to odour under realistic field conditions, and that allows high-throughput screening of many compounds at the same time [3,26].

For lures to be practical, they must be perceived by a high proportion of target insects in the deployment zone where non-directional foraging behaviour (termed kinesis) switches to directional orientation towards an attractive stimulus (positive taxis) or away from a repellent stimulus (negative taxis) [4,9,27]. Since most haematophagous insects feed on highly mobile and often dispersed hosts, some have developed the ability to detect odours from both short (0-20 m) and longer distances (>20 m) [2], but their effective ranges of detection in natural environments are as yet mostly unknown [25]. However, such information is highly relevant for creating efficient synthetic odour blends that, in conjunction with traps and insecticides, lure and kill disease vectors or long-range repellents to protect humans and livestock, and for answering basic biological questions about odour attractiveness and repellency over long distances.

The aim of this study was to test a new methodology that can quantify responses of mosquitoes to odour under realistic field conditions and allow high-throughput screening of many compounds. We used taxis boxes - an assay consisting of three-chambered netting boxes that allows mosquito responses to be studied under field conditions - to investigate the attraction of laboratory-reared *A. gambiae* s.s. and *A. arabiensis* mosquitoes to three stimuli in two separate experiments. The three stimuli were whole human odour, a synthetic blend of carboxylic acids plus carbon dioxide [28] and carbon dioxide alone. Mosquitoes were placed at four distances up to 100m away from the odour source in both experiments. Two effects were analysed: (1) the proportion of mosquitoes that were activated (i.e. that moved out of the middle chamber as a fraction of all mosquitoes) and (2) the proportion of mosquitoes that showed directional movement (i.e. that moved towards the source of stimulation as a fraction of all activated mosquitoes). We show that taxis boxes can be used successfully in a field setting to test the attractiveness of stimuli. Our results also provide insight into the natural range of attraction of *A. gambiae* s.s. and *A. arabiensis,* which may have implications for the design of malaria control strategies and will add to our understanding of olfactory behaviour in these medically important vector species.

## Methods

### Mosquito rearing

Mosquitoes were *Anopheles gambiae* sensu stricto (Ifakara strain, from Njage village, 1996) and *A. arabiensis* (Ifakara strain, from Sacamaganga, 2008). Although *A. gambiae* s.s. has been the predominant malaria vector in sub-Saharan Africa, highly successful indoor vector control has resulted in a changing population composition with *A. arabiensis* filling the niche of *A. gambiae* s.s. in Tanzania [29]. *Anopheles arabiensis* are more adaptable, more likely to bite outdoors and divert to non-human hosts than their specialist anthropophilic siblings [30,31], giving them an important role in maintaining malaria transmission.

Mosquito larvae were maintained at a density of 200 larvae/litre and were fed 0.2mg of ground Tetramin® (Tetra, Melle, Germany) fish food daily. Adult *A. gambiae* s.s. were maintained at 27°C, 70-90% relative humidity and a photoperiod of 12:12 hours light:dark; *A. arabiensis* adults were reared in individual mesh cages under ambient conditions within a netting-enclosed semi-field system at the Ifakara Health Institute (IHI) in southern Tanzania [32]. All adults had constant access to 10% glucose solution and were blood-fed on a human arm every three days.

### Taxis boxes

**Figure 1:**
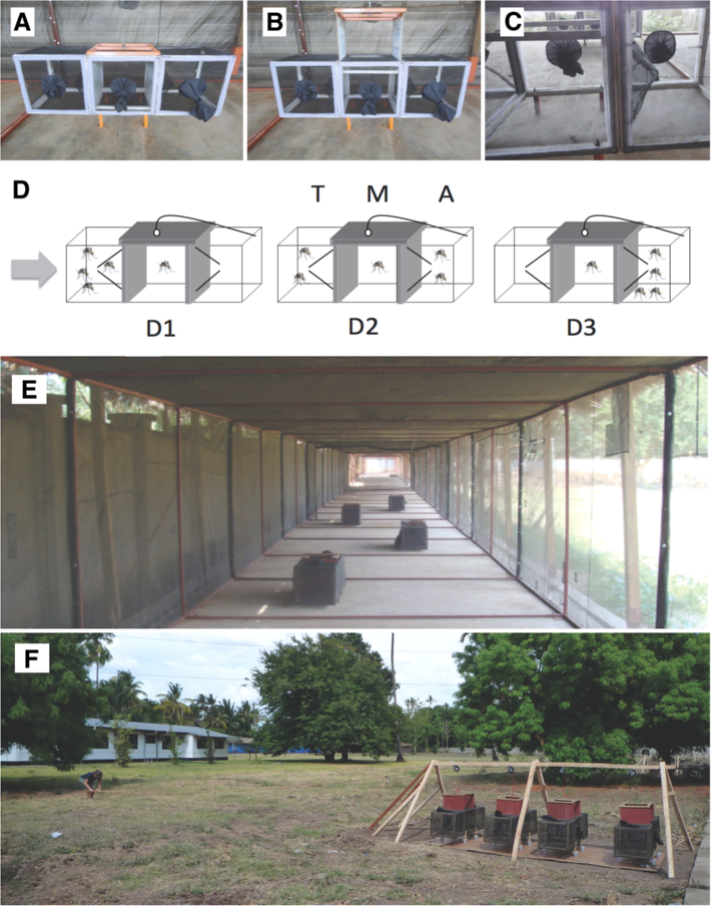
**Experimental set-up. A**) Closed taxis box, and **B**) taxis box in an open position. Mosquitoes were placed in the middle chamber and remained there until a pulley mechanism was used to lift the barriers, allowing access to the two side chambers. **C**) Due to the design of the entry points, mosquitoes could easily fly into a side chamber, but could not return to the centre. Taxis boxes containing mosquitoes exhibiting **D1**) positive taxis (majority move towards (T) the stimulus), **D2**) kinesis (mosquitoes distributed randomly between the three chambers), and **D3**) negative taxis (majority move away (A) from the stimulus) in response to a directional olfactory stimulus indicated by the arrow. **E**) Taxis boxes within the semi-field tunnel, and **F**) four taxis boxes set up in parallel at each distance for the full open field experiment in Ifakara, Tanzania. The human experimenter opens and closes the barriers separating the three chambers by a pulley mechanism to allow mosquitoes to orient towards or away from the point of stimulation.

The assay consisted of taxis boxes (Figure 1A-1C), which are constructed of metal frames overlaid with PVC coated woven fibreglass mosquito netting (TENTEX®). Four wooden legs raise each taxis box 15 cm off the floor and each leg is placed in a cup of water to prevent ants and other crawling insects entering the taxis boxes. Each taxis box consists of three chambers measuring 40 × 40 × 40 cm each: one facing towards the stimulus, one facing away from the stimulus with a central chamber in-between (Figure 1D). The chambers are separated by metal barriers that can be opened and closed by a simple pulley mechanism attached to a rope, to minimise interference to the mosquitoes by the human experimenter (Figure 1F). Mosquitoes are placed into the middle chamber that opens into the two outer chambers by means of two netting funnels tapering to 30 × 2.5 cm. Mosquitoes can fly out of the middle chamber (M) towards (T) or away (A) when the barriers are opened, but cannot return to the middle. This allows the measurement of both effects of interest, namely the proportion of activated mosquitoes and the proportion of directed movement (of the activated mosquitoes). Figure 1D shows mosquitoes exhibiting D1) positive taxis, D2) kinesis (mosquitoes distributed randomly between the chambers), and D3) negative taxis in response to a directional olfactory stimulus indicated by the arrow.

### Stimuli

Each night, one of the three chosen stimuli known to be attractants for *A. gambiae* sensu lato (500 ml min^-1^ CO_2_[33], synthetic blend + 500 ml min^-1^ CO_2_[28], or one human volunteer) or a control (no stimulus) was placed under an untreated bed net at point zero. Odours were dispersed using nylon strips via an MMX counter-flow geometry trap (American Biophysics Corp., East Greenwich, RI), with the trap’s down draft fan used to dispense the odour [22,34]. The blend consisted of hydrous solutions of ammonia (2.5%) and L-lactic acid (85%), propionic acid (C3) at 0.1%, butanoic acid (C4) at 1%, pentanoic acid (C5) at 0.01%, 3-methylbutanoic acid (3mC4) at 0.001%, heptanoic acid (C7) at 0.01%, octanoic acid (C8) at 0.01%, tetradecanoic acid (C14) at 0.01% [28]. Both the MMX trap and the human volunteer remained under an untreated bed net for the duration of the experiment to equalize visual stimuli and the shape of the odour plume. The human volunteers were confirmed to be malaria-negative, wore long trousers and a jacket, and had not bathed for 12 hours prior to the experiment.

### Ethics statement

All human volunteers were informed of the study objectives and risks involved in the experiment, after which they signed a consent form. Ethical approval for the study was obtained from Ifakara Health Institute Institutional Review Board and the Tanzanian National Institute of Medical Research (NIMR) (NIMR/HQ/R.8a/Vol. IX/1255).

### Methodology

Two separate experiments were conducted in Ifakara, Tanzania to test the range of attraction of anopheline mosquitoes. Experiment 2, in which *A. arabiensis* mosquitoes were tested in an open field environment, built upon and extended Experiment 1, where *A. gambiae* s.s. responses were studied in a semi-field system. *Anopheles arabiensis* mosquitoes replaced *A. gambiae* s.s. in the field study because of their increasing importance in residual malaria transmission in the area.

### Experiment 1: semi-field tunnel

Experiment 1 was conducted in Ifakara in a screened semi-field system (semi-field tunnel [SFT], Figure 1E), measuring 110 m long, 2 m wide and 2.5 m high. The SFT is supported by a 2.5 cm steel frame overlaid with TENTEX® and a traditional palm leaf thatch roof. The floor of the structure is concrete with an external water-filled moat to prevent the invasion of ants. The SFT has two entry points – one at each end of the tunnel, both with double entry doors that seal with a zip to prevent mosquitoes escaping from the structure. The tunnel is open to ambient conditions, although airflow is minimised by a wall built along one side. Wind meter (Bioquip) measurements show directional airflow within the tunnel to be between 0 and 2 metres/minute, with occasional air turbulences due to the open nature of the netting. The assay to test the range of mosquito olfaction consisted of 1) a stimulus based at point zero, and 2) a single taxis box at each of four distances (20 m, 50 m, 70 m and 100 m). Four replicates were conducted for each of the three treatments and for the control totalling 16 nights spread over four consecutive weeks (n=4 per treatment). The same volunteer was used for each human replicate.

Nulliparous female *A. gambiae* s.s. mosquitoes (3–8 days old) were sugar starved for four hours prior to the experiment. At 17:00, 50 female mosquitoes were transferred into the middle chamber of each taxis box to settle for 90 minutes prior to the start of the experiment, thus allowing any human odour to dissipate. The stimulus dispenser (MMX) was turned on (or the volunteer sat in the SFT) at 18:00 for 30 minutes prior to the barriers being opened at 18:30 (sunset). Mosquitoes were then given two hours to orient towards or away from the stimulus, and at 20:30 the barriers were replaced to prevent further mosquito movement. The following morning, aspirators were used to remove mosquitoes from the chambers. The mosquitoes were anaesthetised and the number in each chamber of each box was recorded.

### Experiment 2: open field

Experiment 2 was conducted outdoors; a 100 metre-long stretch of vacant land within the IHI site in Ifakara was used for the experiment. The stimulus was placed in the west, with the taxis boxes stretching out towards an easterly direction. A weather station (ProWeatherStation™, Tycon Power Systems, Draper, UT) situated approximately 100 m north of the experimental set up recorded environmental variables such as wind speed and wind direction every 30 minutes.

The assay to test the range of mosquito olfaction consisted of 1) a stimulus based at point zero, and 2) four taxis boxes at each of four distances (10 m, 30 m, 70 m and 100 m; Figure 1F). Each taxis box received an individual ID number and remained in the same position throughout the duration of the experiment. Constraints within the field site limited the placement of the taxis boxes; thus the discrepancy with distances of Experiment 1. Each treatment and the control were tested four times (i.e. over four nights), with four taxis boxes at each distance (n = 16 per treatment). Four different human volunteers were used to test responses to whole body odour to represent natural variation in attractiveness between individuals.

Nulliparous female *A. arabiensis* mosquitoes (3–8 days old) were sugar-starved for 4 hours prior to the experiment. At 18.30, 30 mosquitoes were placed into the middle chamber of each taxis box and allowed to settle for one hour prior to the opening of the barriers at 19.30. Each night, one of the three chosen stimuli or a control (no stimulus) was placed under an untreated bed net and activated at 19.00. At 19.30, the barriers were lifted and the mosquitoes were allowed to orientate for two hours. The barriers were shut at 21.30 to avoid further mosquito movement during the night. The changes in protocol design between Experiments 1 and 2 were due to improved study design (4 replicate boxes at each distance), and practical considerations (the field site was less frequented by people after 19.30). The following morning, mosquitoes were collected from each of the three chambers of each taxis box with aspirators. They were anaesthetised and counted in the laboratory.

### Statistical analysis

Taxis boxes allow mosquitoes to move either towards or away from the experimental stimulus, or to remain in the central chamber. We therefore calculated two response variables from our data. First, we analysed whether the proportion of mosquitoes activated (i.e. moving out of the central chamber, *M*) differed between the stimuli and the control. The response variable - proportion of mosquitoes activated, *a* - was defined as the sum of the number moving towards the stimulus, *T*, and the number moving away, *A*, over the total number of mosquitoes present in the taxis boxes:

$$a=\frac{\left(A+T\right)}{\left(A+M+T\right)}$$

The second analysis tested our main hypothesis that taxis boxes could detect directional movement of mosquitoes in response to olfactory stimuli. From the numbers of mosquitoes that had moved into the chamber closer to the stimulus (number towards, *T*) and the numbers moving into the chamber further from the stimulus (number away, *A*) we defined the second response variable - proportion of mosquitoes attracted to the stimulus, *t* - as

$$t=\frac{T}{\left(T+A\right)}$$

Our statistical analyses were carried out in R version 2.13.0 [35]. The response variables representing activation and taxis were both proportions and our predictor variables included a combination of fixed and random effects. We therefore fitted a generalised linear mixed effects model with binomial errors and the logit link function to the taxis box data using the glmer function from the lme4 package [36]. All models included the variables ‘stimulus’, ‘distance’ and their interaction as fixed effects, and ‘day’ and ‘taxis box ID’ as random effects.

In order to examine the possibility of a non-linear effect of distance, two alternative models were fitted for each dataset in which distance was modelled as either a continuous (i.e. linear) or a categorical (i.e. non-linear) variable. Each model also incorporated two random effects – one for ‘day’ and a second for ‘taxis box ID’ – to reflect the structure of the data and to allow random environmental variability over days and variability between individual boxes to be separated from the effects of our experimental treatments.

Prior to model selection, it was confirmed that the candidate models produced an adequate fit to the data by visually inspecting diagnostic plots of the residuals. For each dataset, the alternative models incorporating linear and non-linear distance relationships were then compared to one another using Akaike Information Criterion (AICc) values [37]. Finally, the significance of individual treatment effects compared to the reference level of the control treatment at each distance was established with Wald z-tests (α = 0.05) calculated from the best-fitting models using the glht function from the multcomp package [38].

## Results

### Experiment 1: semi-field tunnel

A summary of the models fitted in the study is provided as additional material (Additional file 1: Table S1). The best-fitting models incorporated a linear relationship between distance and response for both activation (linear, AICc = 155.1; non-linear, AICc = 170.4) and taxis data (linear, AICc = 147.1; non-linear, AICc = 165.1). Full details of these models are presented in the additional files (see Additional file 1: Tables S2 and Table S3 for tests of the significance of each of the terms in the models and Additional file 1: Tables S6 and Table S7 for the estimated parameter values).

In the semi-field tunnel, the proportion of mosquitoes that moved out of the central chamber of the taxis boxes (i.e. that were activated) in response to stimuli ranged from 34% (67/200) to 53% (106/199) depending on stimulus and distance compared to 25% (53/215) and 39% (80/203) during control nights, at 20m and 100m respectively (Table 1). When measuring taxis, i.e. directional movement of *A. gambiae* s.s. in the SFT, between 31% (22/72) and 36% (19/53) of mosquitoes consistently moved to the *T* chamber of the taxis boxes during control nights regardless of distance (Table 1).

**Table 1:** Mosquito activation and taxis in the semi-field

**Treatment**	**Distance**	**Total**	**Activated i.e. responded**		**Towards**	
		**n**	**n**	%*	**n**	%**
**Control**	**20 m**	215	53	25%	19	36%
	**50 m**	200	59	30%	21	36%
	**70 m**	199	72	36%	22	31%
	**100 m**	203	80	39%	27	34%
**CO**_**2**_	**20 m**	212	96	45%	56	58%
	**50 m**	198	77	39%	50	65%
	**70 m**	201	89	44%	37	42%
	**100 m**	199	106	53%	32	30%
**Blend**	**20 m**	203	99	49%	62	63%
	**50 m**	192	91	47%	57	63%
	**70 m**	210	84	40%	43	51%
	**100 m**	200	67	34%	29	43%
**Human**	**20 m**	211	96	46%	54	56%
	**50 m**	202	86	43%	42	49%
	**70 m**	210	110	52%	38	35%
	**100 m**	198	87	44%	17	20%

* Of total ** of total activated.

Number and proportion of female *Anopheles gambiae* s.s. responding to stimulation (i.e. flying out of the middle chamber) and flying towards the point of stimulation (negative control and three stimuli) obtained from four replicates at four distances in the semi-field tunnel (Experiment 1). n indicates the number of mosquitoes, % indicates the proportion of mosquitoes.

**Figure 2:**
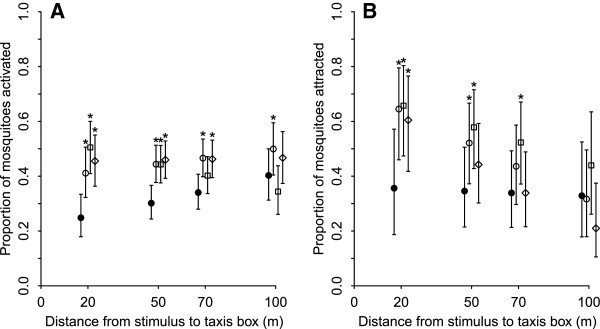
**Activation (A) and taxis movement (B) of A. *gambiae s.s. *towards stimuli in the semi-field tunnel.** Model-estimated proportion of *A. gambiae s.s.* mosquitoes **A**) moving out of the middle chamber either towards or away from the point of stimulation (= activation) or **B**) moving towards the point of stimulation (= taxis) by distance for control nights, i.e. no stimulus (filled circle), 500 ml min^-1^ CO_2_ (open circle), synthetic odour blend + 500 ml min^-1^ CO_2_ (open square) and a human volunteer (open diamond) in the semi-field tunnel (Experiment 1). Each point represents the model-estimated proportion from the best-fitting model, with the associated 95% confidence intervals [59] indicated by vertical bars. NB. At each distance, the data points for the four treatments are slightly offset from one another to improve clarity.

At 20 m, CO_2_, the synthetic blend and the whole human odour all activated and attracted significantly more *A. gambiae* s.s. mosquitoes compared to the control (Table 2; Figure 2). All three stimuli were more than 20% more attractive than the control at 20 metres away (Table 1; Figure 2B). Whereas CO_2_ alone and the synthetic blend elicited a significant activation and attraction response at 50 m, mosquitoes were only activated by – but not attracted to – the human at this distance (Table 2). At 50m, CO_2_ and the synthetic blend were 65% and 63% more attractive, respectively, compared to 36% in the control (Figure 2). At 70 m, CO_2_ and the whole human odour still activated significantly more mosquitoes than the control. However, the blend was the only stimulus that still attracted more mosquitoes at 70 metres distance (Figure 2B). At the furthest distance tested, 100 m, only CO_2_ activated more mosquitoes (53% compared to 39% in control), but none of the stimuli were still more attractive to mosquitoes than the control (Table 2; Figure 2).

**Table 2:** General linear hypothesis tests on mosquito responses in the semi-field tunnel

**Null hypothesis**	**Estimate of difference**	**Standard error of difference**	**z**	**p**
**A) Activation**				
at 20 m:				
**CO**_**2 **_**= Control**	**0.747**	**0.199**	**3.749**	**<0.001**
**Blend = Control**	**1.126**	**0.200**	**5.634**	**<0.001**
**Human = Control**	**0.927**	**0.199**	**4.670**	**<0.001**
at 50 m:				
**CO**_**2 **_**= Control**	**0.614**	**0.142**	**4.319**	**<0.001**
**Blend = Control**	**0.609**	**0.143**	**4.278**	**<0.001**
**Human = Control**	**0.677**	**0.142**	**4.781**	**<0.001**
at 70 m:				
**CO**_**2 **_**= Control**	**0.525**	**0.140**	**3.740**	**<0.001**
Blend = Control	0.265	0.141	1.873	0.061
**Human = Control**	**0.511**	**0.140**	**3.646**	**<0.001**
at 100m:				
**CO**_**2 **_**= Control**	**0.392**	**0.1942**	**2.017**	**0.044**
Blend = Control	−0.252	0.1967	−1.281	0.200
Human = Control	0.261	0.1940	1.344	0.179
**B) Attraction**				
at 20 m:				
**CO**_**2 **_**= Control**	**1.189**	**0.390**	**3.052**	**0.002**
**Blend = Control**	**1.243**	**0.389**	**3.194**	**0.001**
**Human = Control**	**1.017**	**0.389**	**2.610**	**0.009**
at 50 m:				
**CO**_**2 **_**= Control**	**0.722**	**0.307**	**2.349**	**0.019**
**Blend = Control**	**0.953**	**0.307**	**3.104**	**0.002**
Human = Control	0.406	0.307	1.323	0.186
at 70 m:				
CO_2_ = Control	0.411	0.301	1.364	0.173
**Blend = Control**	**0.760**	**0.304**	**2.496**	**0.013**
Human = Control	−0.001	0.305	−0.004	0.997
at 100 m:				
CO_2_ = Control	−0.057	0.369	−0.153	0.878
Blend = Control	0.470	0.381	1.234	0.217
Human = Control	−0.612	0.385	−1.589	0.112

Tests of the general null hypothesis that there is no difference between A) the activation response of *A. gambiae* s.s. mosquitoes and B) the attraction response to each stimulus and to the controls at each distance tested in the semi-field tunnel experiment (Experiment 1), performed using the best-fitting model. Comparisons highlighted in bold are statistically significant at the α = 0.05 level.

### Experiment 2: open field

The recorded wind directions varied between 13° (NNE) and 135° (NWW), but on ten out of the 16 days the wind came from 80° to 120° (S to SWW). However, on 69% (11/16) of the days, the average wind speed was too low to be measured during the hours of 18.00 and 22.00 (output = 0 km/h). On the remaining days, wind speeds ranged from 0.16 to 0.45 km/h during the experimental period. Similarly, the gust levels were 0 km/h on most days (10/16), with fluctuations between 0.51 and 2.77 km/h on the other days.

As for the tunnel experiment, the best-fitting models again incorporated a linear relationship between distance and response for both activation (linear, AICc = 537.0; non-linear, AICc = 541.0) and taxis data (linear, AICc = 462.6; non-linear, AICc = 469.0). Further details for the selected model are presented in the additional material (see Additional file 1: Tables S4 and Table S5 for tests of the significance of each of the terms in the models and Additional file 1: Tables S8 and Table S9 for the estimated parameter values).

**Figure 3:**
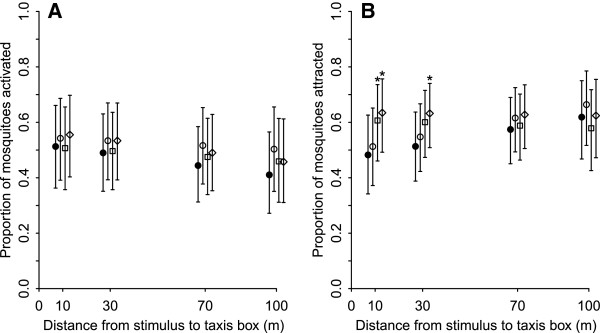
**Activation (A) and taxis movement (B) of A. *gambiae s.s. *towards stimuli in the semi-field tunnel.** Model-estimated proportion of *A. arabiensis* mosquitoes **A**) moving out of the middle chamber either towards or away from the point of stimulation (= activation) or **B**) moving towards the point of stimulation (= taxis) by distance for control nights, i.e. no stimulus (filled circle), 500 ml min^-1^ CO_2_ (open circle), synthetic odour blend + 500 ml min^-1^ CO_2_ (open square) and a human volunteer (open diamond) in the open field environment (Experiment 2). Each point represents the model –estimated proportion from the best-fitting model, with the associated 95% confidence intervals [57] indicated by vertical bars. NB. At each distance, the data points for the four treatments are slightly offset from one another to improve clarity.

In the field, activation levels of female *A. arabiensis* were generally higher than in the SFT and ranged between 41% (189/466) and 57% (234/410) (Table 3). However, there were no statistically significant effects of stimulus or distance on mosquito activation (Table 4; Figure 3A).

**Table 3:** Mosquito activation and taxis in the open field

**Treatment**	**Distance**	**Total**	**Activated i.e. responded**		**Towards**	
		**n**	**n**	%*	**n**	%**
**Control**	**10 m**	392	219	56%	110	50%
	**30 m**	363	158	44%	80	51%
	**70 m**	466	189	41%	105	56%
	**100 m**	438	198	45%	124	63%
**CO**_**2**_	**10 m**	461	258	56%	153	59%
	**30 m**	413	204	49%	98	48%
	**70 m**	447	221	49%	135	61%
	**100 m**	394	209	53%	146	70%
**Blend**	**10 m**	395	222	56%	145	65%
	**30 m**	391	155	40%	77	50%
	**70 m**	410	187	46%	111	60%
	**100 m**	398	194	49%	112	58%
**Human**	**10 m**	410	234	57%	158	68%
	**30 m**	425	215	51%	122	57%
	**70 m**	498	232	47%	139	60%
	**100 m**	382	186	49%	118	63%

* Of total ** of total activated.

Number of female *Anopheles arabiensis* responding to stimulation (i.e. flying out of the middle chamber) and flying towards the point of stimulation (negative control and three stimuli) obtained from four replicates at four distances in the open field (Experiment 2). n indicates the number of mosquitoes, % indicates the proportion of mosquitoes.

**Table 4:** General linear hypothesis tests on mosquito responses in the open field

**Null hypothesis**	**Estimate of difference**	**Standard error of difference**	**z**	**p**
**A) Activation**				
at 10 m:				
CO_2_ = Control	0.119	0.288	0.412	0.680
Blend = Control	−0.025	0.289	−0.086	0.932
Human = Control	0.170	0.288	0.588	0.556
at 30 m:				
CO_2_ = Control	0.176	0.278	0.632	0.528
Blend = Control	0.025	0.279	0.089	0.929
Human = Control	0.174	0.278	0.627	0.531
at 70 m:				
CO_2_ = Control	0.290	0.276	1.048	0.295
Blend = Control	0.123	0.276	0.447	0.655
Human = Control	0.184	0.276	0.667	0.505
at 100 m:				
CO_2_ = Control	0.375	0.291	1.292	0.197
Blend = Control	0.198	0.290	0.680	0.496
Human = Control	0.191	0.290	0.660	0.509
**B) Attraction**				
at 10 m:				
CO_2_ = Control	0.121	0.249	0.486	0.627
**Blend = control**	**0.503**	**0.254**	**1.984**	**0.047**
**Human = control**	**0.621**	**0.251**	**2.472**	**0.013**
at 30 m:				
CO_2_ = Control	0.138	0.228	0.605	0.545
Blend = Control	0.354	0.231	1.534	0.125
**Human = control**	**0.489**	**0.229**	**2.132**	**0.033**
at 70 m:				
CO_2_ = Control	0.171	0.227	0.755	0.450
Blend = Control	0.056	0.228	0.245	0.807
Human = Control	0.222	0.227	0.975	0.330
at 100 m:				
CO_2_ = Control	0.197	0.262	0.750	0.454
Blend = Control	−0.168	0.263	−0.640	0.522
Human = Control	0.022	0.264	0.083	0.934

Tests of the general null hypothesis that there is no difference between A) the activation response of *A. arabiensis* mosquitoes and B) the attraction response to each stimulus and to the controls at each distance tested in the open field experiment (Experiment 2), performed using the best-fitting model. Comparisons highlighted in bold are statistically significant at the α = 0.05 level.

At 10m, significantly more *A. arabiensis* were attracted to the whole human odour and the synthetic blend than to the control with 68% (158/234) and 65% (145/222) moving towards the stimulus respectively, compared to 50% (110/219) for the control. At 30m, the human still attracted significantly more mosquitoes than the control (57% (122/215) compared to 51% (80/158), but neither CO_2_ nor the synthetic blend elicited any response. Beyond 30m, no stimulus tested had any effect on *A. arabiensis* attraction (Table 4; Figure 3B).

## Discussion

In our experiments, we have validated a new behavioural assay – taxis boxes – that allows high-throughput and replicated testing of insect responses to olfactory cues under natural environmental conditions. The behaviour of flying insects towards, or away from, olfactory sources can determine the choice of control strategy used against vectors of medical and veterinary importance. Often, new odours (attractants or repellents) are only developed and tested in laboratories at short ranges and under controlled but mostly artificial conditions. While the value of these studies is unquestionable, more methodologies that allow testing of long-distance olfactory behaviour under naturally variable field conditions are important. We believe that we have developed such a methodology, and show how is can be used to measure the distances over which mosquitoes are able to detect potential blood meal sources and differentiate between stimuli in field settings. Experiment 1 showed that *A. gambiae* s.s. responses to three positive stimuli (CO_2_, synthetic odour and human) can be detected and recorded using taxis boxes in a semi-field tunnel over distances beyond 50 metres. Experiment 2 built upon these findings by repeating the experiment in a natural field setting with *A. arabiensis* females and increasing the replication at each distance to four boxes. This way, we were able to control for environmental variation as well as variability between experimental boxes, measurement error and any potential intrinsic biases within boxes.

Many studies discuss the need for directional airflow when measuring olfactory response in mobile insects [39]. The wind flow in both our experiments was minimal; thus we assume that the current studies relied upon passive diffusion of odour cues in accordance with mathematical simulations of chemotactic searching strategies, which show that the same search algorithm leading an organism to an attractive source can be applied in the presence and absence of wind [40,41]. When we used taxis boxes in an open area with limited vegetation, no airflow or turbulence were detected on more than half of the nights during the time of the study. However, we did not use sensitive wind detectors next to, or at the same height as, the taxis boxes. As the direction and speed of air movement varies with parameters such as height above the ground or type and intensity of vegetation coverage (e.g. [42]), such precise measures of the local environment are essential when trying to understand the mechanism of olfactory-based mosquito movement. This investigation was beyond the scope of our study, but repeating the current work with localised wind detectors would greatly improve our understanding of the mechanistic basis of the role of wind in directional long-distance behaviour.

The first step of host-seeking is the mosquitoes’ activation from resting to an appetitive searching flight, a non-directional movement, which aims to maximise the rate of encounters with attractive host stimuli while reducing costs such as energy expenditure and predation. It is partly driven by the circadian rhythm of species, leading to spontaneous activity before biting [43], but is also affected by external stimuli. In particular, CO_2_ has been shown to be a powerful activator of several mosquito species, getting female mosquitoes excited for a potential blood meal [33,44]. In the SFT, all three stimuli activated more mosquitoes compared to the control up to 50 m, and CO_2_ even up to 100 m. Here, the chemical stimuli are funnelled in the tunnel and are likely to remain more concentrated when reaching mosquitoes compared to the open field. In the open field (Experiment 2), mosquito activation was around 50% regardless of treatment (i.e. whether control or stimuli) or distance from the source of stimulation (Figure 3A). The natural environment is expected to be more saturated with CO_2_ and other attractive odours than the SFT, so that mosquitoes do not require additional stimulus sources to induce their searching flight and blood meal seeking behaviour. Thus, taxis boxes reliably picked up activation and non-directional movement of mosquitoes out of the middle chamber.

However, to study the actual long-distance host-seeking behaviour of mosquitoes beyond activation, directional flight towards attractive stimuli is the key. The taxis boxes allowed us to measure attraction to various stimuli at different distances by comparing the proportion of mosquitoes in the *T* chamber over all mosquitoes that became activated. In the SFT, a higher proportion of mosquitoes were attracted to all three stimuli than during control nights at the closest distance of 20 metres, and the synthetic odour blend remained significantly more attractive up to 70 metres. The assays conducted in the open field (Experiment 2) picked up the mosquitoes’ responses to two of the three stimuli at 10 metres, the closest distance measured, where significantly more females were attracted to the synthetic blend (released in combination with CO_2_) and human odour than when there was only carbon dioxide or no stimulus. As in the semi-field tunnel, this attraction decreased with distance from the source of stimulation with only the human odour attracting more mosquitoes at 30m and no stimulus eliciting a measurable mosquito response at 70 metres.

These distances compare well with previous estimates, for example the attraction of *A. melas* to 500–700 ml min^-1^ CO_2_ or two calves ranged between 18 and 55m [24,25], and did not exceed 30 metres for *A. ziemanni* females [23]. For other insects, the range of odour attraction to a single ox is estimated to be 30-80 m for some horse-fly species [45] and 90 m for *Glossina pallidipes* tsetse flies [46]. Of significance, the current assay allowed differentiation in the range of attraction of the three stimuli with just 16 nights of data collection in contrast to the 60 nights collection performed in the aforementioned field studies. Each taxis box contains an equal number of mosquitoes of the same physiological status and sensitivity at each distance, thus avoiding difficulties encountered in field tests where trap size and efficiency is a great source of bias [47-49]. This gives a strong merit for using them in further studies investigating olfaction in insects.

Carbon dioxide plays an important role in mosquito host-finding and blood-feeding [33,50], although the strength of attraction to CO_2_ depends on a species’ ecological preference and host specialisation. Generalist feeders, such as *A. quadriannulatus* and *A. arabiensis*, have shown higher CO_2_ sensitivities than specialists like *A. gambiae* s.s. [51]. In our studies, *A. gambiae* s.s. showed a strong attraction to CO_2_ in the SFT, whereas, surprisingly, *A. arabiensis* females were not significantly attracted to carbon dioxide in the open field. One explanation could be that carbon dioxide alone is more difficult to detect in natural environments than in the semi-field system. Thus, in the field, mosquitoes will be exposed to a richer mixture of competing semiochemicals and an environment saturated with CO_2_, so that mosquitoes require additional stimuli as well as carbon dioxide for directional flight [2]. In addition, in the open field, mosquitoes may have been affected by the wealth of additional CO_2_ sources from the surrounding environment, causing a confounding effect and thereby not allowing the detection of directional movement, also suggested by the activation analysis. When synthetic or human odours were present, on the other hand, they would have overruled the attractive effect of ambient CO_2_ and caused taxis towards them.

Similarly, the differences in attraction to the synthetic blend and whole human odour between the SFT and open field also suggest that the constantly changing environmental conditions (e.g. moon light, temperature differences) and unmeasured factors, such as the movements of humans and animals in the vicinity of the experiment are less important in determining mosquito host-seeking behaviour in the semi-field system than in the open field. Thus, laboratory-produced odours were more attractive for a longer distance than whole human odour when the blend was less diluted by the environment in the semi-field tunnel. In their natural environment, on the other hand, female anophelines were better able to detect humans for a longer distance. Mosquitoes have evolved specialised odorant receptors in antennae and maxillary palpi [6], which would facilitate the specific selection of suitable host odours, especially from environments crowded with visual and chemical stimuli [52,53]. Selective females would thus maximise their life-time reproductive fitness by balancing energetically-expensive flight with a higher probability of a nutritious blood meal as a reward.

These findings lead to important considerations for malaria vector control. Manipulation of host-seeking and blood-feeding behaviours with odour-baited traps, often treated with insecticide, has successfully been used in tsetse control [54,55] and trialled for control of outdoor-biting malaria vectors [12,14,56]. OBTs could also replace human landing catches as sampling tools to monitor natural arthropod populations, protecting volunteers from exposure to endemic diseases [57]. Most OBTs are baited with synthetically produced blends, which are initially developed and tested in laboratories or wind tunnels at short-ranges. It is therefore essential to identify and understand the natural mode of action of these compounds, and answer whether or not they lure mosquitoes over long distances in enclosed, semi-enclosed and open environments. This will provide information on their optimum placement in village settings, as the preferences of mosquitoes towards particular stimuli can be dependent on the distances between those stimuli [11]. A longer range of attraction would maximise the chances that a resource seeking insect will encounter a given stimulus plume [58]. Thus, efficacy is maximised and disease transmission reduced. Importantly for lure-and-kill or mass trapping strategies, fewer traps would be required per given area, thus reducing per unit cost per resource protected [7,10]. Such findings should be included in models to determine not only the optimum placement of traps for maximum protection against vectors, but also to calculate cost-effectiveness of disease control strategies [7]. In Tanzania, when the same synthetic odour was tested in experimental huts in a full field setting, three- to five-times more *A. arabiensis* mosquitoes were attracted to experimental huts with the synthetic odour compared to humans alone when they were placed 10–100 metres apart. However, when both odour sources directly competed within one hut, the blend became equally or less attractive, increasing the exposure of humans to mosquitoes and potential malaria transmission [28]. In Experiment 1 in the SFT, *A. gambiae* s.s. were attracted two- and three-times more to the synthetic odour than to a human at distances of 70 and 100 metres, respectively. However, in our field experiment, the synthetic blend did not attract more mosquitoes compared to the control beyond 10 metres. In Okumu *et al.*[28], odours were released inside of experimental huts, leading to a different odour plume structure escaping from the eave gap of the huts compared to the open field setting (Experiment 2). Thus, we show that not only the chemical composition of odours but also the structure within which they are released can affect their range of attractiveness to mosquitoes and therefore the choice of malaria control strategy. Taking OBTs as an example, those that are to be used outdoors are likely to require different lure baits than those to be applied inside houses.

## Conclusion

Mosquito host-seeking behaviour occurs in a series of stages, from an appetitive searching flight triggered by internal physiological signals, host detection by volatile semiochemicals to directed flights to the attractive host, landing, probing and biting [2]. In addition to long-distance attraction, taxis boxes could also be used to measure mosquito short-distance responses and compare attraction and repellent efficacies of different stimuli in order to elucidate the relative importance of, for example, odour, carbon dioxide, humidity, temperature or visual cues at close-range host selection. While investigations of malaria vector ecology are important in their own right in developing new evolutionarily sustainable vector control methods and monitoring tools of mosquito populations, taxis boxes have the potential to be used in many other settings as an experimental strategy to understand the ecology and natural olfactory behaviour of other motile arthropods of medical, veterinary and agricultural significance. We therefore believe that the methods described here can provide a practical, broadly applicable system for high-throughput, cost-effective evaluation of compounds used for the olfactory manipulation of insects.

## Competing interests

The author(s) declare that they have no competing interests.

## Authors’ contributions

LML designed and performed the field experiment, analysed the data and drafted the manuscript. AK and ELT performed the statistical analysis and commented on the manuscript. JDM designed and built the tunnel and taxis boxes. CJM and LS designed and performed the semi-field experiment and helped to draft the manuscript. AM, ES and JL performed the studies. LB, FOO, GFK and WRM helped to conceive the study. SJM conceived and designed the study and taxis boxes, and contributed to the writing of the manuscript. All authors read and approved the final version of the manuscript.

## Supplementary Material

Additional file 1 **Lorenz *et al *“Taxis assays measure directional movement of mosquitoes to olfactory cues” (MS: 1181617498954398).**A series of tables that provide a summary of the statistical models fitted in the two studies. **Table S1** shows the model selection, **Tables S2-S5** show the tests of significance of each of the terms in the models and **Tables S6-S9** show the estimated parameter values. Click here for file
