# Supplementary material for: Taxis assays measure directional movement of mosquitoes to olfactory cues
Source: Parasit Vectors. 2013 May 3;6:131. doi: 10.1186/1756-3305-6-131 (PMC3652730; doi:10.1186/1756-3305-6-131)
Supplement: Additional file 1 — Lorenz et al “Taxis assays measure directional movement of mosquitoes to olfactory cues” (MS: 1181617498954398).A series of tables that provide a summary of the statistical models fitted in the two studies. Table S1 shows the model selection, Tables S2-S5 show the tests of significance of each of the terms in the models and Tables S6-S9 show the estimated parameter values. [file 1756-3305-6-131-S1.docx]

**Additional Files**

**Lorenz *et al* “Taxis assays measure directional movement of mosquitoes to olfactory cues” (MS: 1181617498954398)**

**Table S1**

Summary of the models that were fitted in this study. K indicates the number of parameters, with each random effect variance counted as a single parameter. AICc is Aikaike’s Information Criterion with a second-order correction for small sample sizes. Terms highlighted in bold are the best-fitting models selected.

| Experiment | Response | Response numerator | Response denominator | Distance term | K | AICc |
| --- | --- | --- | --- | --- | --- | --- |
| Semi-field tunnel | Proportion of mosquitoes activated, *a* | Number of mosquitoes retrieved from towards and away chambers, *T+A* | Total number of mosquitoes retrieved from all chambers, *T+M+A* | **Linear** | **10** | **155.1** |
|  |  |  |  | Non-linear | 18 | 170.4 |
|  | Proportion of mosquitoes attracted to the stimulus, *t* | Number of mosquitoes retrieved from towards chamber, *T* | Number of mosquitoes retrieved from towards and away chambers, *T+A* | **Linear** | **10** | **147.1** |
|  |  |  |  | Non-linear | 18 | 165.1 |
| Field | Proportion of mosquitoes activated, *a* | Number of mosquitoes retrieved from towards and away chambers, *T+A* | Total number of mosquitoes retrieved from all chambers, *T+M+A* | **Linear** | **10** | **537.0** |
|  |  |  |  | Non-linear | 18 | 541.0 |
|  | Proportion of mosquitoes attracted to the stimulus, *t* | Number of mosquitoes retrieved from towards chamber, *T* | Number of mosquitoes retrieved from towards and away chambers, *T+M+A* | **Linear** | **10** | **462.6** |
|  |  |  |  | Non-linear | 18 | 469.0 |

**Table S2**

Analysis of deviance table calculated using Type II Wald χ^2^ tests for the best-fitting model for mosquito activation in the semi-field semi-field tunnel experiment. Terms highlighted in bold are statistically significant at the α = 0.05 level.

| Model term | χ^2^ | df | p |
| --- | --- | --- | --- |
| **Stimulus** | **22.783** | **3** | **<0.001** |
| Distance | 1.090 | 1 | 0.297 |
| **Stimulus:Distance** | **25.571** | **3** | **<0.001** |

**Table S3**

Analysis of deviance table calculated using Type II Wald χ^2^ tests for the best-fitting model for mosquito taxis in the semi-field tunnel experiment. Terms highlighted in bold are statistically significant at the α = 0.05 level.

| Model term | χ^2^ | df | p |
| --- | --- | --- | --- |
| **Stimulus** | **8.509** | **3** | **0.037** |
| **Distance** | **24.054** | **1** | **<0.001** |
| **Stimulus:Distance** | **12.395** | **3** | **0.006** |

**Table S4**

Analysis of deviance table calculated using Type II Wald χ^2^ tests for the best-fitting model for mosquito activation in the field experiment. Terms highlighted in bold are statistically significant at the α = 0.05 level.

| Model term | χ^2^ | df | p |
| --- | --- | --- | --- |
| Stimulus | 0.909 | 3 | 0.823 |
| Distance | 2.994 | 1 | 0.084 |
| Stimulus:Distance | 3.112 | 3 | 0.375 |

**Table S5**

Analysis of deviance table calculated using Type II Wald χ^2^ tests for the best-fitting model for mosquito taxis in the field experiment. Terms highlighted in bold are statistically significant at the α = 0.05 level.

| Model term | χ^2^ | df | p |
| --- | --- | --- | --- |
| Stimulus | 2.537 | 3 | 0.469 |
| Distance | 1.699 | 1 | 0.192 |
| **Stimulus:Distance** | **13.297** | **3** | **0.004** |

**Table S6**

Parameter estimates for the best-fitting model for mosquito activation in the semi-field tunnel experiment. The standard deviations of the random effects terms were estimated to be: Day = 0.126; Box = 0.000. Parameters highlighted in bold are statistically significant at the α = 0.05 level.

| Parameter | Estimate | Standard error | z | p |
| --- | --- | --- | --- | --- |
| **Intercept** | **-1.284** | **0.188** | **-6.831** | **<0.001** |
| *Stimulus* |  |  |  |  |
| **CO_2_** | **0.836** | **0.255** | **3.273** | **0.001** |
| **Blend** | **1.471** | **0.257** | **5.731** | **<0.001** |
| **Human** | **1.094** | **0.255** | **4.297** | **<0.001** |
| **Distance** | **0.009** | **0.003** | **3.453** | **0.001** |
| *Stimulus:Distance* |  |  |  |  |
| CO_2_:Distance | -0.004 | 0.004 | -1.256 | 0.209 |
| **Blend: Distance** | **-0.017** | **0.004** | **-4.816** | **<0.001** |
| **Human:Distance** | **-0.008** | **0.004** | **-2.360** | **0.018** |

**Table S7**

Parameter estimates for the best-fitting model for mosquito taxis in the semi-field tunnel experiment. The standard deviations of the random effects terms were estimated to be:: Day = 0.345; Box = 0.114. Parameters highlighted in bold are statistically significant at the α = 0.05 level.

| Parameter | Estimate | Standard error | z | p |
| --- | --- | --- | --- | --- |
| Intercept | -0.561 | 0.389 | -1.442 | 0.149 |
| *Stimulus* |  |  |  |  |
| **CO_2_** | **1.501** | **0.474** | **3.168** | **0.002** |
| **Blend** | **1.436** | **0.475** | **3.021** | **0.003** |
| **Human** | **1.424** | **0.477** | **2.985** | **0.003** |
| Distance | -0.002 | 0.005 | -0.304 | 0.761 |
| *Stimulus:Distance* |  |  |  |  |
| **CO_2_:Distance** | **-0.016** | **0.006** | **-2.657** | **0.008** |
| Blend: Distance | -0.010 | 0.006 | -1.600 | 0.110 |
| **Human:Distance** | **-0.020** | **0.006** | **-3.324** | **0.001** |

**Table S8**

Parameter estimates for the best-fitting model for mosquito activation in the field experiment. The standard deviations of the random effects terms were estimated to be: Day = 0.375; Box = 0.239. Parameters highlighted in bold are statistically significant at the α = 0.05 level.

| Parameter | Estimate | Standard error | z | p |
| --- | --- | --- | --- | --- |
| Intercept | 0.098 | 0.235 | 0.416 | 0.677 |
| *Stimulus* |  |  |  |  |
| CO_2_ | 0.090 | 0.295 | 0.306 | 0.759 |
| Blend | -0.049 | 0.296 | -0.167 | 0.867 |
| Human | 0.167 | 0.295 | 0.566 | 0.571 |
| **Distance** | **-0.005** | **0.002** | **-2.059** | **0.039** |
| *Stimulus:Distance* |  |  |  |  |
| CO_2_:Distance | 0.003 | 0.002 | 1.396 | 0.163 |
| Blend: Distance | 0.002 | 0.002 | 1.207 | 0.228 |
| Human:Distance | 0.000 | 0.002 | 0.118 | 0.906 |

**Table S9**

Parameter estimates for the best-fitting model for mosquito taxis in the field experiment. The standard deviations of the random effects terms were estimated to be: Day = 0.265; Box = 0.281. Parameters highlighted in bold are statistically significant at the α = 0.05 level.

| Parameter | Estimate | Standard error | z | p |
| --- | --- | --- | --- | --- |
| Intercept | -0.132 | 0.228 | -0.577 | 0.564 |
| *Stimulus* |  |  |  |  |
| CO_2_ | 0.112 | 0.264 | 0.426 | 0.670 |
| **Blend** | **0.578** | **0.269** | **2.146** | **0.032** |
| **Human** | **0.688** | **0.267** | **2.579** | **0.010** |
| **Distance** | **0.006** | **0.003** | **2.133** | **0.033** |
| *Stimulus:Distance* |  |  |  |  |
| CO_2_:Distance | 0.001 | 0.003 | 0.292 | 0.770 |
| **Blend: Distance** | **-0.007** | **0.003** | **-2.533** | **0.011** |
| **Human:Distance** | **-0.007** | **0.003** | **-2.260** | **0.024** |
